# Variability Attribution for Automated Model Building

**DOI:** 10.1208/s12248-019-0310-5

**Published:** 2019-03-08

**Authors:** Moustafa M. A. Ibrahim, Rikard Nordgren, Maria C. Kjellsson, Mats O. Karlsson

**Affiliations:** 1 Department of Pharmaceutical Biosciences, Uppsala University, Uppsala, Sweden; 2 Department of Pharmacy Practice, Helwan University, Cairo, Egypt

**Keywords:** automated model building, linearization, model evaluation, nonlinear mixed effects models, stochastic model

## Abstract

We investigated the possible advantages of using linearization to evaluate models of residual unexplained variability (RUV) for automated model building in a similar fashion to the recently developed method “residual modeling.” Residual modeling, although fast and easy to automate, cannot identify the impact of implementing the needed RUV model on the imprecision of the rest of model parameters. We used six RUV models to be tested with 12 real data examples. Each example was first linearized; then, we assessed the agreement in improvement of fit between the base model and its extended models for linearization and conventional analysis, in comparison to residual modeling performance. Afterward, we compared the estimates of parameters’ variabilities and their uncertainties obtained by linearization to conventional analysis. Linearization accurately identified and quantified the nature and magnitude of RUV model misspecification similar to residual modeling. In addition, linearization identified the direction of change and quantified the magnitude of this change in variability parameters and their uncertainties. This method is implemented in the software package PsN for automated model building/evaluation with continuous data.

**Electronic supplementary material:**

The online version of this article (10.1208/s12248-019-0310-5) contains supplementary material, which is available to authorized users.

## INTRODUCTION

Nonlinear mixed effect (NLME) modeling, commonly known as the population approach, is increasingly used to describe longitudinal data from preclinical/clinical experiments, either to improve the efficiency of the drug development process and subsequent dosing, or increase the understanding of the studied underlying pathophysiological system (1). In contrast to naive pooling approach, which ignores individual differences, and two-stage approach, which does not distinguish between subject and observation variability, NLME models allow pooling of sparse data from different subjects while simultaneously quantifying multiple levels of variability, thanks to its mixed effects nature. In mixed-effects analysis, population parameters are included in a model as fixed effects, and the variability within this population as random effects. Random effects can incorporate variability on both the subject and observation levels, as inter-individual variability (IIV), between occasion variability, between study variability, and residual unexplained variability (RUV). This ability to identify different sources of variability is particularly critical to many clinical applications, e.g., therapeutic drug monitoring.

For highly nonlinear models, extending the structural base model to include covariates or test different models for random effects can be tedious and interrupted by numerical difficulties. These problems increase exponentially with increasing the complexity of the structure, covariate, and variability models. To overcome such computational and time-intensive burden, linear approximation of first-order conditional estimation (FOCE) method was proposed and applied as a diagnostic tool for testing covariates and random effects (2,3). When successfully implemented, linearization substantially reduced runtimes compared to standard NLME models as the fixed effects are not estimated in the linearized models, but fixed to their estimates from the fit of the NLME model. Linearized models were shown to result in similar objective function values (OFVs) to the NLME models, and accurately identify significant covariate relations and stochastic components similar to conventional analysis. Hence, linearization output models in a standardized coding format, linearization was also recommended for automated model building by coupling to other covariate modeling algorithms as stepwise covariate method (SCM) or full random effects covariate modeling (FREM) (4). However, linearized models still need to be estimated given the original observations similar to NLME models, so it might be sensitive to local minima or other estimation-related issues, especially in presence of interactions between empirical Bayes estimates and RUV models. Major deviations between the OFV of the linearized structure base model and its corresponding NLME model should be interpreted as a failure of implementation of linearization and must be solved prior to further investigations using the linearized model. It has not been shown previously that random effects estimated in linearized models or their uncertainties’ have similar values if estimated in the corresponding NLME models, which if true, will support the role of linearization in automated model building to predict changes in random variability assigned to model parameters upon the inclusion of a potential covariate or adoption of a new RUV model.

Meanwhile, a new method “residual modeling” was proposed as a fast and robust diagnostic tool for assessing RUV models for NLME analysis with continuous outcomes (5). Residual modeling treats the outputted residuals from a NLME model execution as a dependent variable to model its distribution’s mean and variance by a linear base model, then this base model is extended to assess different RUV extensions. The improvement in the fit between the residuals base model and its extended versions can accurately identify the nature and magnitude of potential RUV model improvements/misspecifications, and hence, residual modeling has been already implemented for automated model building. Residual modeling uses a built-in library of six RUV extensions to model the variance of the residuals’ distribution from a NLME model execution. The built-in library includes autoregressive (AR1), dynamic transform both sides (dTBS), residuals’ IIV, power, t-distribution, and time-varying RUV models (6–10). The investigated residuals were conditional weighted residuals (CWRES), conditional weighted residuals with interaction (CWRESI), individual weighted residuals (IWRES), and normalized prediction distribution errors (NPDE); CWRES outperformed the rest, as CWRES modeling correctly identified the type of the needed RUV model and accurately predicted both the estimates of parameters governing this RUV model and the magnitude of improvement of fit after implementing such RUV model. Residual modeling does not suffer from local minima problems or estimation related issues, as it is using residuals data, not the original observations. This is an advantage for its purpose in fast and robust selection of the best RUV model, but then by definition, it cannot predict the impact of implementing a new RUV model on random variability assigned to the rest of model parameters or their uncertainties.

Here, we investigated if linearization can predict variability attribution for automated model building on the inclusion of a new RUV extension. We used the same six RUV models from our previous work for residual modeling. (5) First, we compared the performance of linearization to residual modeling in selecting the best RUV extension; then, we compared random effects’ estimates and uncertainties on linearized models with the different RUV extensions to their corresponding NLME models.

## METHODS

### Linearization

For continuous outcome, let *y*_*ij*_ be the observation *j* for individual *i*, *θ* is the vector of population parameters, *η*_*i*_ is the vector of unexplained deviation of individual parameters *θ*_*i*_ from the population parameters *θ*, *x*_*ij*_ is the vector of individual *i* design components as dose and sampling times, and *ε*_*ij*_ is the residual error of observation *j* for individual *i*, then the NLME model describing the observations:

1 $$ {y}_{ij}=f\left(\theta, {\eta}_i,{x}_{ij}\right)+h $$

where *f* is model prediction, and *h* is the RUV model to be function of *ε*_*ij*_. Such model can be extended further for multivariate outcome, baseline or time varying covariates. Both random effects *η*_*i*_ and *ε*_*ij*_ are assumed to follow normal distribution with mean 0 and covariance matrix Ω and Σ, respectively, and the unknown model parameters are estimated by maximum likelihood. According to the way of the dependence of *h* on *f*, this NLME model can be linearized based on first-order Taylor expansion around *ε*_*ij*_ = 0 and the empirical Bayes estimate ${\widehat{\eta}}_i$:

2 $$ {y}_{ij}\approx f\left(\theta, {\widehat{\eta}}_i,{x}_{ij}\right)+{f}^{\prime}\left(\theta, {\widehat{\eta}}_i,{x}_{ij}\right)\left({\eta}_i-{\widehat{\eta}}_i\right)+{h}^{\prime}\left({\varepsilon}_{ij}-0\right)+\frac{\partial {h}^{\prime }}{\partial {\eta}_i}\left({\varepsilon}_{ij}-0\right)\left({\eta}_i-{\widehat{\eta}}_i\right) $$

3 $$ {f}_{ij}=f\left(\theta, {\widehat{\eta}}_i,{x}_{ij}\right)+{f}^{\prime}\left(\theta, {\widehat{\eta}}_i,{x}_{ij}\right)\left({\eta}_i-{\widehat{\eta}}_i\right) $$

4 $$ {h}_{ij}={\varepsilon}_{ij}\ \left({h}^{\prime }+\frac{\partial {h}^{\prime }}{\partial {\eta}_i}\left({\eta}_i-{\widehat{\eta}}_i\right)\right) $$

5 $$ {y_{ij}}^{\ast }={f}_{ij}+{h}_{ij} $$

where *y*_*ij*_^∗^ is the linearized model, *f*_*ij*_ is the approximated individual predictions, and *h*_*ij*_ is the approximated individual residual errors.

The NLME model is first evaluated to calculate the different partial derivatives and ${\widehat{\eta}}_i$ needed, then *y*_*ij*_^∗^ is estimated on the same dataset of the NLME model to obtain *η*_*i*_ and *ε*_*ij*_, as these are the only unknown parameters in *y*_*ij*_^∗^. With estimating only random effects alongside its standard coding format, *y*_*ij*_^∗^ can be easily and quickly used as a base model for further explaining *η*_*i*_ with covariates or using different *ε*_*ij*_ models (3). Here, we extended (Eq. 5) to test six RUV models, and compare their goodness of fit, parameters’ variability estimates, and uncertainties to conventional testing by NLME models as follow and shown in the Supplementary material.

### RUV extensions

To test the dependence of *ε*_*ij*_ at time point *j* on *ε*_*ik*_ at time point k, *autoregression* (AR1) *error model* with one extra parameter can be implemented:

6 $$ \rho \left({\varepsilon}_{ij},{\varepsilon}_{ik}\right)={\mathrm{e}}^{-\left(\ln (2)/{t}_{1/2}\right)\left({time}_j-{time}_k\right)} $$

where *ρ* is the correlation between these errors and *t*_1/2_ is the half-life of *ρ*. The improvement of fit after implementing AR1 error model in the linearized model (∆OFV_*lin*, *AR*1_) is calculated as the difference in OFV of the linearized base model (Eq. 5) and OFV of the linearized model with AR1 error model (Eqs. 5 and 6). ∆OFV_*lin*, *AR*1_ is comparable to the improvement of fit on implementing AR1 error model in the NLME model (∆OFV_*NLME*, *AR*1_) between the base NLME model (Eq. 1) and its AR1 error model extension.

In presence of skewness in residuals distribution, *dynamic transform both sides* (dTBS) approach is useful through the estimation of a Box–Cox shape parameter *λ* and a power term *ζ* that also address possible scedasticity in residual magnitudes (8,9). Linearized models with dTBS approach follows (Eq. 7) if *λ* was estimated to 0, and (Eq. 8) otherwise. Improvement of fit on dTBS implementation (∆OFV_*lin*, *dTBS*_) is the difference in OFVs of the dTBS linearized model with *λ* and *ζ* fixed to 1 and 0, respectively, and the dTBS linearized model with both *λ* and *ζ* estimated.

7 $$ \ln \left({y_{ij}}^{\ast}\right)=\ln \left({f}_{ij}\right)+{h}_{ij}\bullet {f_{ij}}^{\zeta } $$

8 $$ \frac{{{y_{ij}}^{\ast}}^{\lambda }-1}{\lambda }=\frac{{f_{ij}}^{\lambda }-1}{\lambda }+{h}_{ij}\bullet {f_{ij}}^{\zeta } $$

One of maximum likelihood assumptions regarding the residual error *ε*_*ij*_ is being identically distributed, this assumption can be relaxed by adding *inter-individual variability η*_*i*, *RUV*_ *on the residuals* to allow different RUV magnitudes. Improvement of such extension in the fit of the linearized model (∆OFV_*lin*, *IIV*_) is the difference in OFVs of (Eq. 5) and (Eq. 9).

9 $$ {y_{ij}}^{\ast }={f}_{ij}+{h}_{ij}\bullet {e}^{\eta_{i, RUV}} $$

In absence of skewness, the dependence of residuals magnitude on model predictions can be corrected with *ζ* alone in what is known as *power RUV model*. Improvement of fit on applying the power RUV model to the linearized models (∆OFV_*lin*, *ζ*_) is the difference in OFVs of (Eq. 5) and (Eq. 10).

10 $$ {y_{ij}}^{\ast }={f}_{ij}+{h}_{ij}\bullet {f_{ij}}^{\zeta } $$

Assuming normal distribution of residuals means that large errors do not exist, which if not true will force maximum likelihood estimation to shift model parameters’ estimates to fulfill small errors assumption. This bias can be avoided by introducing *t-distributed residuals*. The Laplacian method with user-defined conditional likelihood (*L*) had to be used for a Laplace linearized base model (Eq. 11) and linearized model with t-distributed residuals (Eq. 12), where *σ* is the square root of *h*_*ij*_, and *υ* is the degrees of freedom; the difference of these models’ OFVs is ∆OFV_*lin*, *υ*_.

11 $$ L=\left(1/\sqrt{2\pi {\sigma}^2}\right)\exp \left(-\frac{1}{2}{\left(\frac{{y_{ij}}^{\ast }-{f}_{ij}}{\sigma}\right)}^2\right) $$

12 $$ L=\frac{\varGamma \left(\frac{\upsilon +1}{2}\right)}{\ \varGamma \left(\frac{\upsilon }{2}\sqrt{\upsilon \pi {\sigma}^2}\right)}{\left(1+\frac{1}{\upsilon }{\left(\frac{{y_{ij}}^{\ast }-{f}_{ij}}{\sigma}\right)}^2\right)}^{-\left(\frac{\upsilon +1}{2}\right)} $$

Lastly, *time-varying errors* allow different error magnitudes for different time points. A typical example is that the absorption phase in a pharmacokinetic model can have larger errors than the elimination phase. This is implemented by allowing the change of the standard deviation of residuals to be a step function of the time or time after dose, at selected cutoff time point *X*.

13 $$ {\displaystyle \begin{array}{c}\omega ={\uptheta}_1\\ {} if\ \left( time>X\right)\kern0.75em \omega ={\uptheta}_2\\ {}{h}_{ij}=\upomega \bullet {\varepsilon}_{ij}\ \left({h}^{\prime }+\frac{\partial {h}^{\prime }}{\partial {\eta}_i}\left({\eta}_i-{\widehat{\eta}}_i\right)\right)\ \end{array}} $$

where θ_1_ is the standard deviation of residuals before the cutoff time point *X*, θ_2_ is the standard deviation of residuals after this cutoff time point, and Σ is fixed to 1, as multiplying a random variable by constant (ω ∙ *ε*_*ij*_) increase the variance by the square of this constant (ω^2^). We used three cutoff points to divide the data into four equal sized groups, and the improvement of fit (∆OFV_*lin*, *time*_) after extending the linearized base model (Eqs. 4 and 5) to the linearized model with time varying residuals (Eqs. 5 and 13) is the difference between their respective OFVs.

### Evaluations

**Fig. 1:**
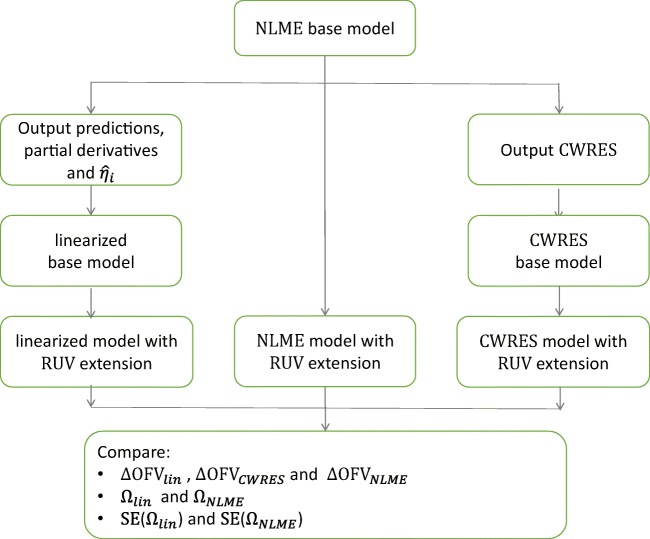
Schematic presentation of the method used to evaluate linearization ability in predicting variability attributions

These extended linearized models (example code in Supplementary material) were estimated to obtain their respective improvement of fit ∆OFV_*lin*_, as well as Ωs’ estimates and uncertainties. We compared the performance of ∆OFV_*lin*_ in predicting ∆OFV_*NLME*_ to that of ∆OFV_*Diagnostic*_ obtained by residual modeling, where *diagnostic* refers to the used residual. Afterwards, we compared Ωs’ estimates and uncertainties of linearized models to their respective NLME models as shown in Fig. 1. We used 12 real data examples for our evaluation (Table I). Only when the linearized base model and the NLME base model had similar OFV were RUV extensions added and further estimated. All real data examples were treated as continuous. Asenapine effects were assessed using PANSS, which is a composite score, where items of positive, negative, and general nature are scored and combined into one assessment. Despite this, the asenapine data was treated as continuous data in the model. Also, the asenapine model was implemented with residuals’ IIV model from the start. Models varied in structure components from simple pharmacokinetic one compartment model as moxonodine, to complex description of nonlinear system of interacting multi-dependent variables as the integrated glucose-insulin (IGI) model. Seven models used log-transformed data. Two models used a combined error model, two models used a proportional error model and the remaining models used additive error models. NONMEM version 7.4.3 (ICON Development Solutions, Hanover, MD, USA) was used for the analysis (22), with the aid of the *linearize* tool in PsN (3,23), and graphs were generated in R (24). To obtain the improvement of fit by residual modeling (∆OFV_*Diagnostic*_) when testing the different RUV extensions on the real data examples, we used the *resmod* tool in PsN (5).

**Table I:** Summary of real data examples used for investigation

Model	Data type	RUV model	Transformation	No. of observations	No. of subjects	No. of THETAs	No. of OMEGAs including covariances	No. of SIGMAs
Asenapine^a^ (11)	PD	Additive with IIV	–	7728	1328	16	5	1
Clomethiazole (12)	PK	Additive	Log	2177	772	10	5	1
Daunorubicin^a^ (13)	PD	Additive	Log	112	41	7	3	1
Digoxin^a,b^ (14)	PK/PD	PD: proportional PK: additive	–	941	225	6	3	1
Disufenton sodium^a^ (15)	PK	Additive	Log	1196	175	7	3	1
Ethambutol^a^ (16)	PK	Combined	Log	1869	189	8	3	1
IGI^ab^ (17)	PD	Additive	Log	6382	72	26	15	1
Miltefosine^a^ (18)	PK	Proportional	–	350	31	7	4	1
Moxonodine^a^ (10)	PK	Additive	Log	1021	74	5	6	1
Paclitaxel^a,c^ (19)	PD	Combined	–	530	46	6	3	1
Pefloxacin^a^ (20)	PK	Additive	Log	337	74	4	6	1
r-Hfsh (21)	PK	Additive	–	314	60	7	2	1

^a^SIGMAs were fixed to 1 and modeled as THETAs (standard deviation)

^b^More than one dependent variable

^c^Additive component of RUV model was fixed

## RESULTS

**Fig. 2:**
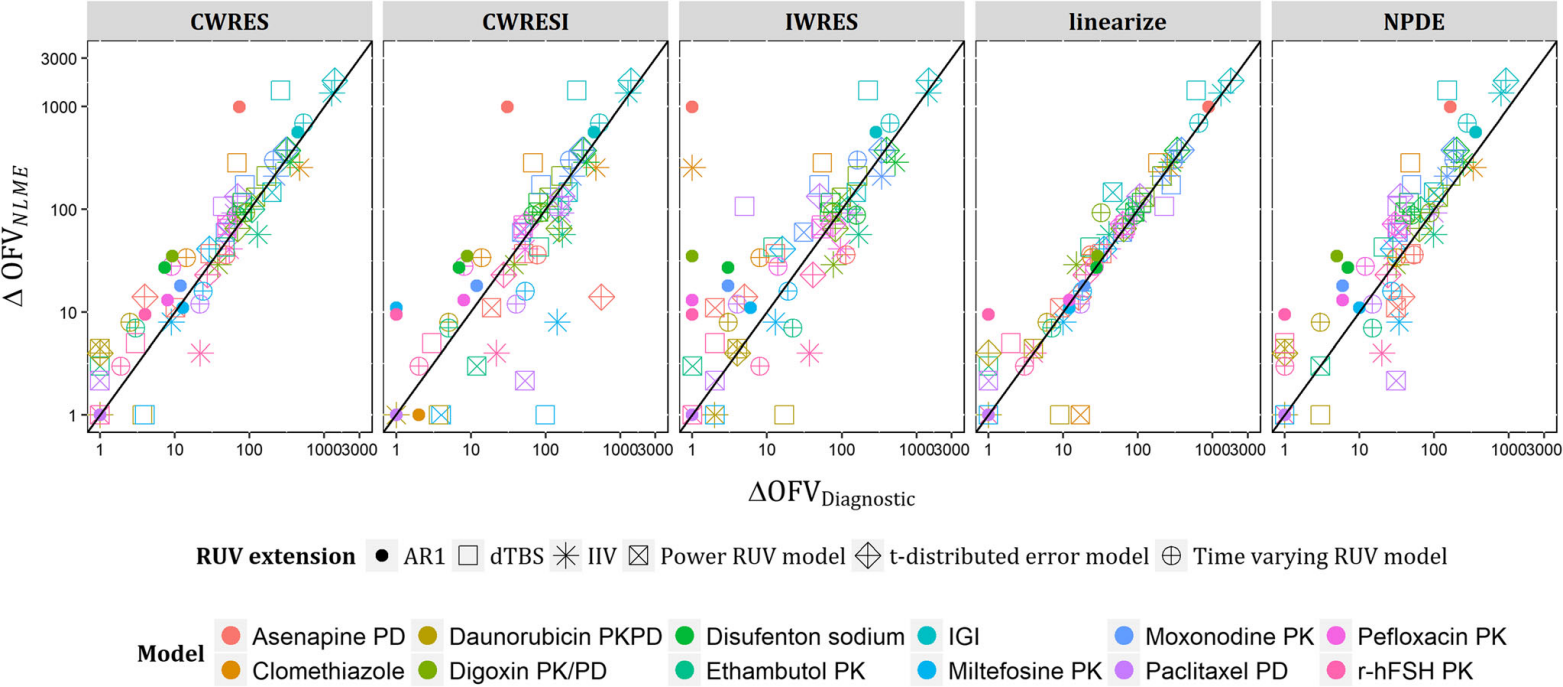
Plot of absolute ΔOFV_*NLME*_versus absolute ΔOFV for CWRES, CWRESI, IWRES, linearization, and NPDE among the real data examples for the six extended RUV models

Linearization was successfully applied to all examples, justified by the similarities in the OFVs of the linearized base models and the NLME base models. All examples were extended successfully to the different RUV models, except for AR1 and t-distribution error models with Clomethiazole and the IGI models. All examples benefitted significantly with one or more of the RUV extensions, except for Daunorubicin model. Across all examples, the agreement between ∆OFV_*lin*_ and ∆OFV_*NLME*_ was good as shown in Fig. 2. Comparing to the performance of residual modeling in predicting ∆OFV_*NLME*_, linearization surpassed CWRESI, IWRES, and NPDE over all different ranges of ∆OFV_*NLME*_, and performed better than CWRES at most ranges of ∆OFV_*NLME*_ except at low ranges of ∆OFV_*NLME*_(~ 10) where CWRES was slightly better. Linearization identified accurately the most important RUV extension to all examples similar to conventional analysis, surpassing CWRES modeling that reversed the order of 1st and 2nd most important extensions with two examples, Ethambutol and Disufenton sodium models. Also, linearization identified the RUV extensions resulting in significant improvement of fit in all examples similar to conventional analysis, while CWRES modeling missed only t-distribution error model with Asenapine model, shown in Fig. 2. Asenapine model is the only model with residuals’ IIV model as the base model, which may be sufficient in explaining outliers and would turn the t-distributed error model rather less important. The median ratio of ΔOFV_*lin*_/ΔOFV_*NLME*_ was 0.95 among models with significant improvement, compared to 0.8 for the median ratio of ΔOFV_*CWRES*_/ΔOFV_*NLME*_.

**Fig. 3:**
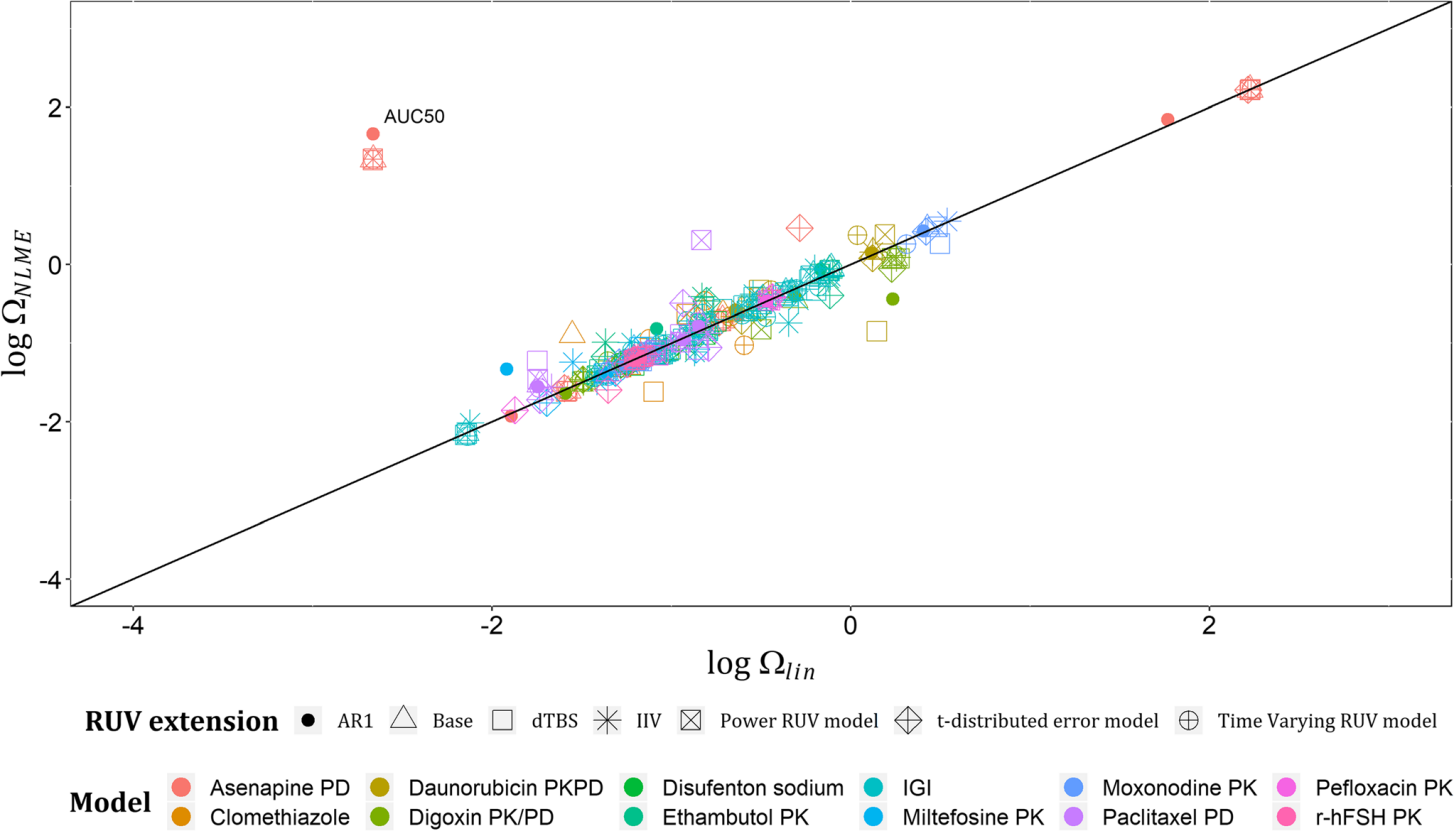
Plot of log (Ω_NLME_) versus log (Ω_lin_) across the real data examples for the six extended RUV models, with only one outlier: the variability assigned to AUC50 parameter in Asenapine model with all RUV extensions except t-distributed error model

**Fig. 4:**
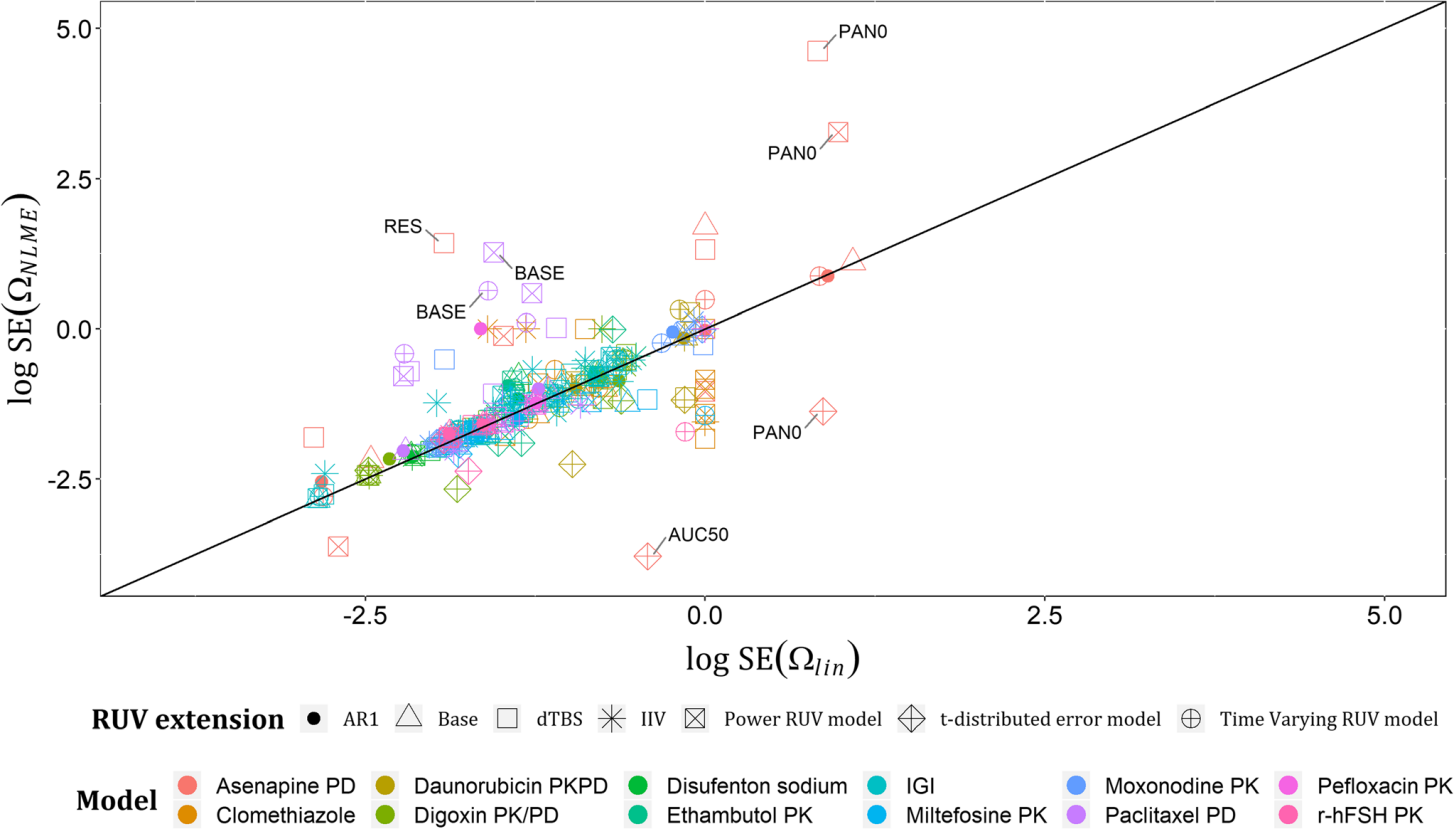
Plot of log SE (Ω_NLME_) versus log SE (Ω_lin_) across the real data examples for the six extended RUV models. Departures (± 2 units from identity line) are the log standard error estimates of the variabilities assigned to PAN0, AUC50 and RES parameters in Asenapine model, and BASE parameter in Paclitaxel model

**Fig. 5:**
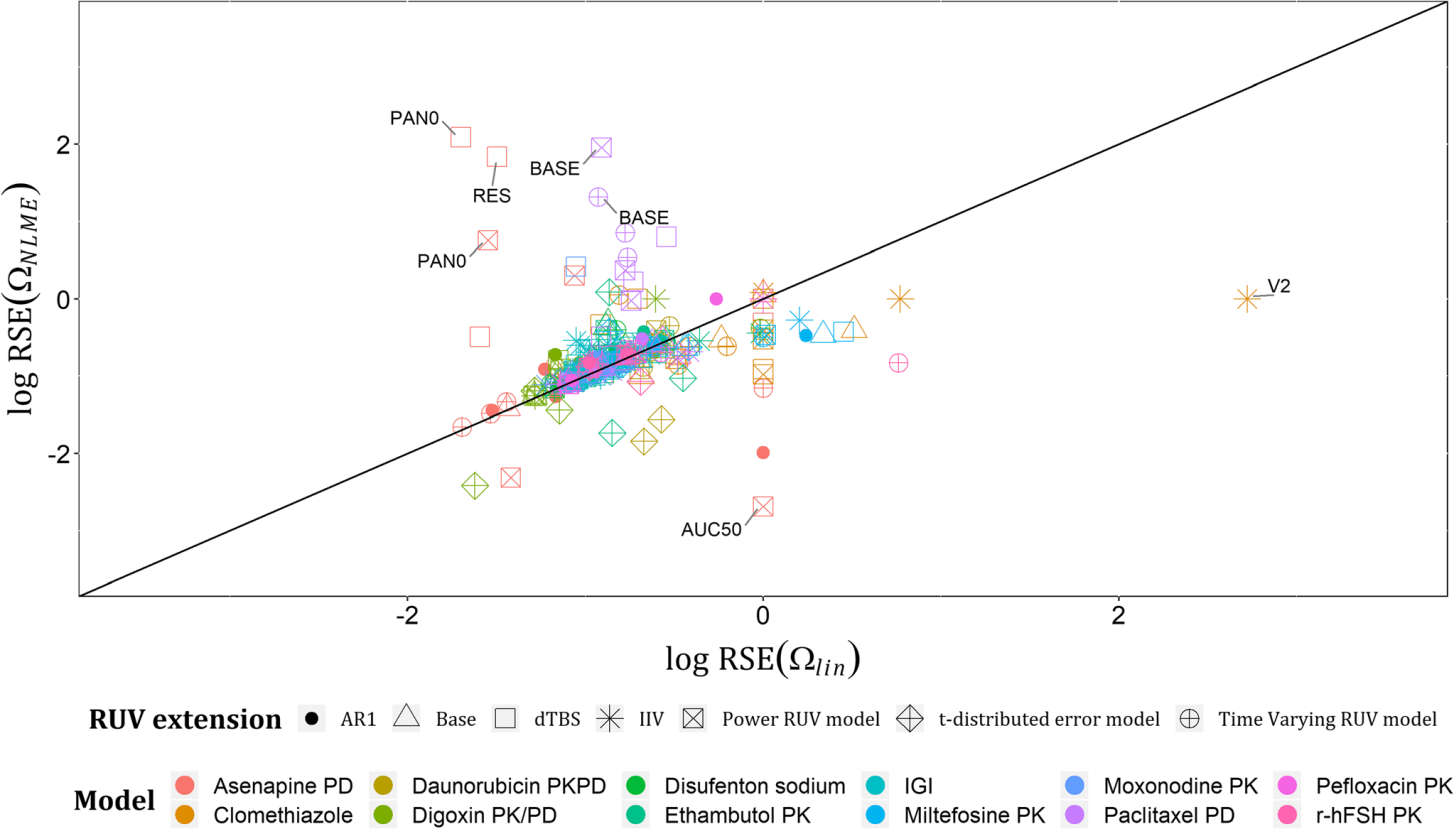
Plot of log RSE (Ω_NLME_) versus log RSE (Ω_lin_) across the real data examples for the six extended RUV models. Departures (± 2 units from identity line) are the log relative standard error estimates of the variabilities assigned to PAN0, AUC50, and RES parameters in Asenapine model, BASE parameter in Paclitaxel model, and V2 parameter in Clomethiazole model

Regarding the estimates of Ωs on linearized models (Ω_*lin*_) and their respective estimates on NLME models (Ω_*NLME*_), they showed good agreement with only one outlier: AUC50’s variability in asenapine model. A plot of log (Ω_*NLME*_) versus log (Ω_*lin*_) across all examples with base models and their RUV extensions is shown in Fig. 3, with estimates less than − 4 on log scale excluded from the graph, given that these estimates are low and would not be considered in further model development. In total, nine estimates were excluded based on this, e.g., the variability assigned to the intercompartmental clearance in Clomethiazole model under IIV on RUV and dTBS extensions. Standard errors (SEs) of each Ω_*lin*_ and its respective Ω_*NLME*_ showed a good agreement in the commonly expected range of SEs for a well identifiable continuous data variability parameter (0–1), and bad agreement at the extreme estimates of SE(Ω_*NLME*_), for instance, the SE of PAN0’s variability in asenapine model was > 1000 with both dTBS and power RUV extensions, which is unacceptable. This may be related to the scores used to measure asenapine effect, i.e., PANSS. A plot of the log-transformed estimates of SE(Ω_*NLME*_) and SE(Ω_*lin*_) is presented in Fig. 4, with estimates less than − 4 on log scale excluded from the graph. Lastly, relative standard errors (RSEs) for each Ω_*lin*_ and its corresponding Ω_*NLME*_ were calculated on the standard deviation scale as (Eq. 14), and their log-transformed estimates are presented in Fig. 5, that in addition to showing the same trends as Fig. 4, showed that standard errors after implementing t-distribution extensions are less predictable by linearization than the other RUV extensions.

14 $$ \mathrm{RSE}\left(\Omega \right)=\frac{\mathrm{SE}\left(\Omega \right)}{\Omega}/2 $$

## DISCUSSION

In this paper, we explored if the use of linearization to identify and quantify RUV model misspecifications, similar to residual modeling (5), can provide additional advantages. Residual modeling assesses whether RUV extensions are required to address an RUV misspecification. It is done in an extremely fast and robust way, thanks to the simple nature of models for residuals data. In case of multiple dependent variables, residual modeling evaluates the RUV extensions separately for each dependent variable, identifying which variable need which extension, and so reducing the risk of ending up with an over-parameterized NLME model. However, being estimated on residual data has shortcomings, as residual modeling cannot inform on the rest of the NLME model parameters. Implementation of a needed RUV extension in a NLME model would be expected to improve the uncertainties of Ω and θ subsequently, as the latter is a function of the former. Linearization, in contrast to residual modeling, uses the calculated parameters’ partial derivatives with respect to ${\widehat{\eta}}_i$ from the fit of the NLME model. It estimates the RUV model incorporating any extension and the random effects components given the same data as the NLME model. Thus, linearization can estimate explicitly the random effects and their uncertainties in the base and the extended model, and implicitly the magnitude and the direction of change in the random effects and their uncertainties, and that is what we had shown here.

We successfully implemented six RUV extensions to the standardized linearization framework and linearized all real data examples. However, estimation difficulties were present when applying AR1 and t-distribution RUV extensions to the NLME/linearized models of Clomethiazole and the IGI, but not in their respective residual modeling. The agreement between ΔOFV_*lin*_ and ΔOFV_*NLME*_ when improvement of fit is > 10 was nearly perfect, indicating that only the estimates of random effects were changing on implementing the different RUV extensions in the NLME models. Deviations would be expected if estimates of fixed effects were also changing. The overall prediction performance of ΔOFV_*NLME*_ by ΔOFV_*lin*_ was better than ΔOFV_*CWRES*_, however not by much.

Linearization identified and quantified the nature and the magnitude of RUV model misspecifications in these real data examples more accurately than CWRES modeling, the latter reversed the order of the most two important extensions in Ethambutol and Disufenton sodium models and could not identify t-distribution as a significant extension in Asenapine model. Even though it is a minor difference, it illustrated the high sensitivity of linearization to detect differences between the RUV extensions that introduce similar flexibility in the model, e.g., IIV and t-distribution RUV models both offer outlier robustness in the NLME model. This showed that conclusions drawn from the results of automated testing of RUV extensions will remain the same on replacement of residual modeling with linearization, and the only expected difference would be an increased run time for linearization of structure models with large random effects models.

Regarding the prediction of the impact of RUV extensions on Ω_*NLME*_, linearization showed a good ability with only one outlier (the Ω assigned to AUC50 in Asenapine model). Interestingly, linearization underestimated this Ω with all RUV extensions except t-distribution. This underestimation issue will escalate when it comes to predicting SE(Ω_*NLME*_). Linearization did well in assessing the expected ranges of uncertainties of variability assigned to model parameters describing continuous data; more deviations occurred as uncertainties’ estimates moved away from that range, with the main problem being Asenapine model. This might point out that violation of assumptions regarding the nature of data will be a problematic in this automated testing procedure as Asenapine effects are measured using PANSS, which is a composite score, but treated as continuous data in the model. Among the RUV extensions, t-distribution was the most associated with deviations, mainly underestimation. That can be easily tracked down to the use of LAPLACE method commonly known for minimization-related problems, for instance, SE(Ω_*NLME*, *υ*_) of AUC50 in Asenapine model was 1.66 × 10^−4^ which is too close to 0, and an unreasonable estimate for uncertainty, given that the estimate of Ω_*NLME*, *υ*_ of AUC50 is 2.9. This problem of unreasonable estimates in SE(Ω_*NLME*_) explain all the extreme deviations seen in Fig. 4. One of these is PAN0 parameter in asenapine model which Ω_*NLME*, *dTBS*_ estimate was 168, but its SE(Ω_*NLME*, *dTBS*_) was 4.15 × 10^4^. With these deviations being justified, it is safe to claim that linearization itself or its predictive performance of SE(Ω_*NLME*_) showed no built-in drawbacks. The same issues go for RSE(Ω_*NLME*_) as not respecting the nature of the data, t-distribution extension, and the unrealistic estimates of Ω_*NLME*_ and their uncertainties propagated to most of the outliers in Fig. 5.

In conclusion, we investigated the possible merits of linearization if used to evaluate RUV models for continuous data. Linearization accurately identified the nature of RUV extension if needed and predicted the improvement of fit on its inclusion similar to residual modeling. In addition, linearization can predict the impact of including such RUV extension on the variability assigned to model parameters and their uncertainties, allowing its utilization for variability attribution with automated model building procedures.

## Electronic Supplementary Material

ESM 1 (PDF 157 kb)
