# Supplementary material for: Variability Attribution for Automated Model Building
Source: AAPS J. 2019 Mar 8;21(3):37. doi: 10.1208/s12248-019-0310-5 (PMC6505507; doi:10.1208/s12248-019-0310-5)
Supplement: Supplementary file 1 — (PDF 157 kb) [file 12248_2019_310_MOESM1_ESM.pdf]

NONMEM code for Linearized models:

1. Linearized base model:

```
$PROB  base model
$INPUT  ID TIME DV OPRED D_EPS1 D_ETA1 D_ETA2 OETA1 OETA2
D_EPSETA1_1 D_EPSETA1_2
$DATA   run_base_linbase.dta  IGNORE=@  IGNORE(MDV.NEN.0)
$PRED
BASE1=D_ETA1*(ETA(1)-OETA1)
BASE2=D_ETA2*(ETA(2)-OETA2)
BASE_TERMS=BASE1+BASE2
IPRED=OPRED+BASE_TERMS
ERR1=EPS(1)*(D_EPS1+D_EPSETA1_1*(ETA(1)-OETA1))
ERR2=EPS(1)*(D_EPSETA1_2*(ETA(2)-OETA2))
ERROR_TERMS=ERR1+ERR2
Y=IPRED+ERROR_TERMS
$OMEGA   0.075 ; IIV CL
$OMEGA   0.056 ; IIV V
$SIGMA   1
$ETAS       FILE=run_base.phi
$ESTIMATION MCETA=1 METHOD=COND INTERACTION MAXEVALS=9999
$COVARIANCE UNCOND
;-------------------------------------------------------------
```

2. Linearized model with autocorrelated errors:

```
$PROB  AR1
$INPUT  ID TIME DV OPRED D_EPS1 D_ETA1 D_ETA2 OETA1 OETA2
D_EPSETA1_1 D_EPSETA1_2
$DATA   run_base_linbase.dta  IGNORE=@  IGNORE(MDV.NEN.0)
$ABBREVIATED DECLARE T(NO)
$ABBREVIATED DECLARE INTEGER I,DOWHILE J
$PRED
IF(NEWIND.NE.2) THEN
I=0
END IF

IF(NEWL2==1.AND.MDV==0) THEN
I=I+1
T(I)=TIME
J=1
DO WHILE (J<=I)
CORRL2(J,1) = EXP((-0.6931/THETA(1))*(TIME-T(J)))
J=J+1
ENDDO
ENDIF
BASE1=D_ETA1*(ETA(1)-OETA1)
BASE2=D_ETA2*(ETA(2)-OETA2)
BASE_TERMS=BASE1+BASE2
IPRED=OPRED+BASE_TERMS
ERR1=EPS(1)*(D_EPS1+D_EPSETA1_1*(ETA(1)-OETA1))
ERR2=EPS(1)*(D_EPSETA1_2*(ETA(2)-OETA2))
ERROR_TERMS=ERR1+ERR2
Y=IPRED+ERROR_TERMS
```

```
$THETA   0.001 ; corr half-life
$OMEGA   0.075 ; IIV CL
$OMEGA   0.056 ; IIV V
$SIGMA   1
$ETAS       FILE=run_base.phi
$ESTIMATION MCETA=1 METHOD=COND INTERACTION MAXEVALS=9999
$COVARIANCE UNCOND
;--------------------------------------------------------------
```

3. Linearized model with dynamic transform both sides (dTBS):

```
$PROB  dtbs model
$INPUT  ID TIME DV OPRED D_EPS1 D_ETA1 D_ETA2 OETA1 OETA2
D_EPSETA1_1 D_EPSETA1_2
$DATA   run_base_linbase.dta  IGNORE=@  IGNORE(MDV.NEN.0)
$SUBROUTINE       CONTR=contr.txt          CCONTR=ccontra.txt
$PRED
ZETA=THETA(1)
LAMBDA=THETA(2)
BASE1=D_ETA1*(ETA(1)-OETA1)
BASE2=D_ETA2*(ETA(2)-OETA2)
BASE_TERMS=BASE1+BASE2
IPRED=OPRED+BASE_TERMS
ERR1=THETA(3)*(D_EPS1+D_EPSETA1_1*(ETA(1)-OETA1))
ERR2=THETA(3)*(D_EPSETA1_2*(ETA(2)- OETA2))
ERROR_TERMS=ERR1+ERR2
W=ERROR_TERMS*(IPRED**ZETA)
IPRTR=IPRED
 IF (LAMBDA .NE. 0 .AND. IPRED .NE.0) THEN
    IPRTR=(IPRED**LAMBDA-1)/LAMBDA
 ENDIF
 IF (LAMBDA .EQ. 0 .AND. IPRED .NE.0) THEN
    IPRTR=LOG(IPRED)
 ENDIF
 IF (LAMBDA .NE. 0 .AND. IPRED .EQ.0) THEN
    IPRTR=-1/LAMBDA
 ENDIF
 IF (LAMBDA .EQ. 0 .AND. IPRED .EQ.0) THEN
    IPRTR=-1000000000
 ENDIF
 IPRED=IPRTR
Y=IPRED + W*EPS(1)
$THETA   0.001 ; ZETA
$THETA   0.001 ; lambda
$THETA   0.33  ; RES ERR
$OMEGA   0.075 ; IIV CL
$OMEGA   0.056 ; IIV V
$SIGMA   1 FIX
$ETAS       FILE=run_base.phi
$ESTIMATION MCETA=1 METHOD=COND INTERACTION MAXEVALS=9999
$COVARIANCE UNCOND
;--------------------------------------------------------------
```

4. Linearized model with Inter-individual variability (IIV) on RUV:

```
$PROB  omega on epsilon
$INPUT  ID TIME DV OPRED D_EPS1 D_ETA1 D_ETA2 OETA1 OETA2
D_EPSETA1_1 D_EPSETA1_2
$DATA   run_base_linbase.dta  IGNORE=@  IGNORE(MDV.NEN.0)
$PRED
BASE1=D_ETA1*(ETA(1)-OETA1)
BASE2=D_ETA2*(ETA(2)-OETA2)
BASE_TERMS=BASE1+BASE2
IPRED=OPRED+BASE_TERMS
ERR1=EPS(1)*(D_EPS1+D_EPSETA1_1*(ETA(1)-OETA1))
ERR2=EPS(1)*(D_EPSETA1_2*(ETA(2)-OETA2))
ERROR_TERMS=ERR1+ERR2
Y=IPRED+(ERROR_TERMS*EXP(ETA(3)))
$OMEGA   0.075 ; IIV CL
$OMEGA   0.056 ; IIV V
$OMEGA   0.01  ; IIV on RUV
$SIGMA   1
$ETAS       FILE=run_base.phi
$ESTIMATION MCETA=1 METHOD=COND INTERACTION MAXEVALS=9999
$COVARIANCE UNCOND
;--------------------------------------------------------------
```

5. Linearized model with power error model:

```
$PROB  power model
$INPUT  ID TIME DV OPRED D_EPS1 D_ETA1 D_ETA2 OETA1 OETA2
D_EPSETA1_1 D_EPSETA1_2
$DATA   run_base_linbase.dta  IGNORE=@  IGNORE(MDV.NEN.0)
$PRED
BASE1=D_ETA1*(ETA(1)-OETA1)
BASE2=D_ETA2*(ETA(2)-OETA2)
BASE_TERMS=BASE1+BASE2
IPRED=OPRED+BASE_TERMS
ERR1=EPS(1)*(D_EPS1+D_EPSETA1_1*(ETA(1)-OETA1))
ERR2=EPS(1)*(D_EPSETA1_2*(ETA(2)-OETA2))
ERROR_TERMS=ERR1+ERR2
SCALE=IPRED**THETA(1)
Y=IPRED+(ERROR_TERMS*SCALE)
$THETA   0.001 ; Power on RUV
$OMEGA   0.075 ; IIV CL
$OMEGA   0.056 ; IIV V
$SIGMA   1
$ETAS       FILE=run_base.phi
$ESTIMATION MCETA=1 METHOD=COND INTERACTION MAXEVALS=9999
$COVARIANCE UNCOND
;--------------------------------------------------------------
```

6. Linearized base model for t-distributed errors:

```
$PROB  t-distribution base model
$INPUT  ID TIME DV OPRED D_EPS1 D_ETA1 D_ETA2 OETA1 OETA2
D_EPSETA1_1 D_EPSETA1_2
$DATA   run_base_linbase.dta  IGNORE=@  IGNORE(MDV.NEN.0)
```

```
$PRED
BASE1=D_ETA1*(ETA(1)-OETA1)
BASE2=D_ETA2*(ETA(2)-OETA2)
BASE_TERMS=BASE1+BASE2
IPRED=OPRED+BASE_TERMS
ERR1=THETA(1)*(D_EPS1+D_EPSETA1_1*(ETA(1)-OETA1))
ERR2=THETA(1)*(D_EPSETA1_2*(ETA(2)- OETA2))
W=ERR1+ERR2
IWRES=(DV-IPRED)/W
LIM=10E-14
IF(IWRES.EQ.0) IWRES=LIM
LL=-0.5*LOG(2*3.14159265)-LOG(W)-0.5*(IWRES**2)
Y=-2*LL
$THETA   0.01  ; RES ERR
$OMEGA   0.075 ; IIV CL
$OMEGA   0.056 ; IIV V
$ETAS       FILE=run_base.phi
$ESTIMATION METHOD=1 MAXEVALS=9999 LAPLACE -2LL
$COVARIANCE UNCOND
;------------------------------------------------------------
```

7. Linearized model with t-distributed errors:

```
$PROB  t-distribution model
$INPUT  ID TIME DV OPRED D_EPS1 D_ETA1 D_ETA2 OETA1 OETA2
D_EPSETA1_1 D_EPSETA1_2
$DATA   run_base_linbase.dta  IGNORE=@  IGNORE(MDV.NEN.0)
$PRED
BASE1=D_ETA1*(ETA(1)-OETA1)
BASE2=D_ETA2*(ETA(2)-OETA2)
BASE_TERMS=BASE1+BASE2
IPRED=OPRED+BASE_TERMS
ERR1=THETA(1)*(D_EPS1+D_EPSETA1_1*(ETA(1)-OETA1))
ERR2=THETA(1)*(D_EPSETA1_2*(ETA(2)- OETA2))
W=ERR1+ERR2
DF=THETA(2)  ; degrees of freedom of t-distribution
IWRES=(DV - IPRED)/W
PHI=(DF + 1)/2
INN=PHI + 1/(12 * PHI - 1 / (10 * PHI))
GAMMA=SQRT(2 * 3.14159265 / PHI) * (INN / EXP(1))**PHI
PHI2=DF/2
INN2= PHI2 + 1 / (12 * PHI2 - 1 / (10 * PHI2))
GAMMA2=SQRT(2*3.14159265/PHI2)*(INN2/EXP(1))**PHI2
COEFF=GAMMA/(GAMMA2*SQRT(DF*3.14159265))/W
BASE=1+IWRES*IWRES/DF  ; base of PDF of t-distribution
POW=-(DF+1)/2          ; power of PDF of t-distribution
L=COEFF*BASE**POW      ; PDF of t-distribution
Y=-2*LOG(L)
$THETA   0.01  ; RES ERR
$THETA  (3,5)  ; DF
$OMEGA   0.075 ; IIV CL
$OMEGA   0.056 ; IIV V
$ETAS       FILE=run_base.phi
$ESTIMATION METHOD=1 MAXEVALS=9999 LAPLACE -2LL
$COVARIANCE UNCOND
;------------------------------------------------------------
```

8. **Linearized model with time varying errors:**

;With example cut-off time points (1.5,2 & 6 hr)

```
$PROB  time varying
$INPUT  ID TIME DV OPRED D_EPS1 D_ETA1 D_ETA2 OETA1 OETA2
D_EPSETA1_1 D_EPSETA1_2
$DATA   run_base_linbase.dta  IGNORE=@  IGNORE(MDV.NEN.0)
$PRED
WA=THETA(1)
IF (TIME.LT.1.5) WA=THETA(2)
IF (TIME.GE.1.5.AND.TIME.LT.2) WA=THETA(3)
IF (TIME.GE.2.AND.TIME.LT.6) WA= THETA(4)
BASE1=D_ETA1*(ETA(1)-OETA1)
BASE2=D_ETA2*(ETA(2)-OETA2)
BASE_TERMS=BASE1+BASE2
IPRED=OPRED+BASE_TERMS
ERR1=(WA*EPS(1))*(D_EPS1+D_EPSETA1_1*(ETA(1)-OETA1))
ERR2=(WA*EPS(1))*(D_EPSETA1_2*(ETA(2)-OETA2))
ERROR_TERMS=ERR1+ERR2
Y=IPRED + ERROR_TERMS
$THETA  (0,.33) ; RES ERR
$THETA  (0,.33)
$THETA  (0,.33)
$THETA  (0,.33)
$OMEGA   0.075 ; IIV CL
$OMEGA   0.056 ; IIV V
$SIGMA   1 FIX
$ETAS       FILE=run_base.phi
$ESTIMATION MCETA=1 METHOD=COND INTERACTION MAXEVALS=9999
$COVARIANCE UNCOND
;--------------------------------------------------------------
```

9. **contr.txt file for dTBS models:**

```
subroutine contr (icall,cnt,ier1,ier2)
double precision cnt
call ncontr (cnt,ier1,ier2,l2r)
return
end
;--------------------------------------------------------------
```

10. **ccontra.txt file for dTBS models:**

```
subroutine ccontr (icall,c1,c2,c3,ier1,ier2)
USE ROCM_REAL,   ONLY: theta=>THETAC,y=>DV_ITM2
USE NM_INTERFACE,ONLY: CELS
double precision c1,c2,c3,w,one,two,lambda
dimension c2(:),c3(:,:)
data one,two/1.,2./
if (icall.le.1) return
w=y(1)
         lambda=theta(2)
         if(lambda.eq.0) y(1)=log(y(1))
         if(lambda.ne.0) y(1)=(y(1)**lambda-one)/lambda
call cels (c1,c2,c3,ier1,ier2)
y(1)=w
c1=c1-two*(lambda-one)*log(y(1))
return
end
```
